# Comparison of Deep and Moderate Neuromuscular Blockade on Intestinal Mucosal Barrier in Laparoscopic Gastrectomy: A Prospective, Randomized, Double-Blind Clinical Trial

**DOI:** 10.3389/fmed.2021.789597

**Published:** 2022-02-02

**Authors:** He Huang, Ling Zhou, Yingying Yu, Shijiang Liu, Hao Xu, Zekuan Xu, Chun Yang, Cunming Liu

**Affiliations:** ^1^Department of Anaesthesiology and Perioperative Medicine, The First Affiliated Hospital of Nanjing Medical University, Nanjing, China; ^2^Department of General Surgery, The First Affiliated Hospital of Nanjing Medical University, Nanjing, China

**Keywords:** gut microbiota, intestinal function, intestinal mucosal barrier, laparoscopic gastrectomy, neuromuscular blockade

## Abstract

Deep neuromuscular blockade (NMB) improves the surgical conditions and is benefit for the postoperative recovery after laparoscopic surgery. However, the mechanisms of deep NMB in promoting the recovery of intestinal function have not been completely investigated. The objective of our study was to determine the impact of the deep NMB and moderate NMB strategy on the intestinal barrier function after laparoscopic gastrectomy. We collected patients undergoing elective laparoscopic gastrectomy. Patients were randomized to deep NMB (post-tetanic count 1–2) vs. moderate NMB (train-of-four count 1–2) during the surgery. Primary outcomes were time to flatus, serum diamine oxidase (DAO) and D-lactate, and gut microbiota. Other outcomes were surgical condition scores, postoperative visual analog pain scores, and length of hospital stay. Ninety patients in deep NMB group and sixty patients in moderate NMB group completed the study. Main results showed that the time to flatus was decreased in deep NMB group (74 ± 32 h) than that in moderate NMB group (93 ± 52 h, *P* = 0.006). The level of serum D-lactate was statistically increased in the moderate NMB group than that in the deep NMB group (1,209 ± 224 vs. 1,164 ± 185 ng/ml, *p* < 0.001). But no significant differences could be detected in the level of DAO between the groups. Additionally, the 16s rRNA analysis indicated that gut microbiota were similar in Alpha diversity but distinct in Beta diversity. Furthermore, the beneficial bacteria, such as genus *Lactobacillus* and *Bifidobacterium*, were more abundant in the deep NMB group, while the potentially harmful bacteria were more abundant in the moderate NMB group. Our findings suggested that the intestinal mucosal barrier and gut microbiota were better preserved in deep NMB, which greatly improved the postoperative recovery of intestinal function after laparoscopic gastrectomy.

## Introduction

The intestinal mucosal barrier, which is composed of mechanical, chemical, microbial, and immunologic barriers, is essential for the normal intestinal function. The integrated intestinal mucosal barrier obstructs the harmful substances and plays a key role in human health and diseases. However, it would be easily damaged in surgeries or trauma with physical, chemical, and biological injuries (1).

Diamine oxidase (DAO) and D-lactate are common biomarkers to identify the intestinal barrier dysfunction. DAO is an enzyme abundant in intestinal mucosa which is localized mainly in the small intestinal mucosa, predominantly in the tips of the villi (2). It is the essential substance for the cell proliferation function and is the oxidative deaminating of several polyamines (3). Once the epithelia of intestinal mucosa were damaged, DAO released from the cells would be absorbed into blood. Thus, the level of serum DAO is a sensitive biomarker for evaluating the integrity of intestinal mucosa (4). D-lactate is another serum biomarker produced by some kinds of gut microbiota, which cannot be metabolized in human. A large amount of D-lactate can enter the circulation through the damaged mucosa when the intestinal mucosal permeability increases (5). With the advent of the genome era, gene sequencing has been widely used to display the microbial diversity. Moreover, 16s rRNA sequencing is one of the methods for the detection of intestinal microbiota at the level of phylum, class, order, family, and genus. Increased bacterial abundance and diversity are the important indicators of intestinal health (6).

Owing to less postoperative complications and faster rehabilitation compared with the traditional open surgery, laparoscopic surgery has become the major surgical approach for elective surgeries (7, 8). However, several factors, such as the surgery methods or the analgesic management, are considered to retard the recovery of intestinal function after laparoscopic surgery. Though the modified approach of the surgery and the multimodal analgesia partly reduce the worries about the intestinal side effects, previous studies have reported that the delayed recovery of bowel movement was associated with the long-lasting laparoscopic process with the continuous insufflation of carbon dioxide (CO_2_) (9, 10).

Deep neuromuscular blockade (NMB) with low intra-abdominal pressure, which is an important element of enhanced recovery after surgery (ERAS) in gastrointestinal surgeries, may be responsible for remitting the postoperative intestinal dysfunction. It has been demonstrated that deep NMB improves the surgical conditions in laparoscopic surgery with a lower demand for CO_2_ insufflation (11). Thus, deep NMB is recommended especially in the laparoscopic surgeries performed in proximity to the diaphragm, such as laparoscopic cholecystectomy or gastrectomy (12, 13).

Given the extensive use of deep NMB in laparoscopic surgeries, it is of great importance to investigate the deep NMB and moderate NMB strategy on the intestinal barrier function and prognosis. We therefore performed this prospective study to compare the effect of different depth of NMB on the intestinal mucosal barrier after laparoscopic gastrectomy surgery with a comparison of the biomarkers assessing the intestinal barrier dysfunction, 16s rRNA sequencing of gut microbiota, and other perioperative parameters.

## Methods

### Study Design and Population

This prospective, randomized, and double-blind study was carried out from January 2019 to March 2021 at the First Affiliated Hospital of Nanjing Medical University. Informed written consent was obtained from each participant before enrolment. The protocol of this study was approved by the Hospital Research Ethics Committee of the First Affiliated Hospital of Nanjing Medical University, Nanjing, China (No: 2018-SR-336). This clinical trial was registered at https://www.clinicaltrials.gov (NCT03782233).

Eligible patients with early- or intermediate-stage gastric cancer met the inclusion criteria: age 40–80 years.; body mass index (BMI) <30 kg/m^2^; American Society of Anesthesiologists (ASA) classification I–III; and scheduled for laparoscopic gastrectomy. Exclusion criteria included: inflammatory bowel diseases or intestinal obstruction; severe heart or lung diseases; severe renal insufficiency or liver diseases; long-term antibiotic therapy before surgery; history of abdominal surgery; neuromuscular diseases (e.g., gravis myasthenia); and allergy to the study medication.

All patients were randomized to either deep NMB group or moderate NMB group at a ratio of 1.5:1. The randomization sequence was produced with a random number generator and sealed with numbered envelopes providing randomized group allocation.

### Study Intervention

The NMB monitor using an acceleromyograph (Mindray BeneView T9, Shenzhen, China) was set at the ulnar nerve for acquiring the train-of-four (TOF) responses or post-tetanic twitches. The deep NMB was maintained with a continuous infusion of rocuronium 0.5–0.6 mg/(kg·h) to a target post-tetanic count (PTC) of 1–2. A continuous infusion of rocuronium 0.2–0.3 mg/(kg·h) was titrated in the moderate NMB group to a target TOF count of 1–2. At the end of surgery, sugammadex 2 mg/kg was administrated intravenously at a TOF count of 2 for NMB reversal.

All surgeries were done by the same surgeon and assistants. Two anesthetists were responsible for anesthesia management. The depth of neuromuscular blockade was adjusted according to the group intervention by the chief anesthetist who was un-blinded to the group allocation, while the assistant, who was blinded to the group allocation, was responsible for recording perioperative outcomes. Both surgeon and patients were blinded to the group allocation.

### Intraoperative Management

All patients were monitored with ECG, pulse oximetry, invasive arterial blood pressure, and bispectral index (BIS). Anesthesia was induced with etomidate 0.3 mg/kg, midazolam 0.02 mg/kg, and fentanyl 3–5 μg/kg. The acceleromyograph was standardized when the patient was asleep. A single bolus of rocuronium 0.6 mg/kg was injected intravenously for intubation. Mechanical ventilation was performed in the volume control mode after intubation with tidal volume 6–8 ml/kg, respiration rate 12–16 times/min, positive end expiratory pressure (PEEP) 5 cmH_2_O, and the air-oxygen mixture of 60% fraction of inspiration O_2_ (F_i_O_2_). Anesthesia was maintained with propofol 1.5 mg/(kg·h), remifentanil 0.03–0.1 μg/(kg·min) and sevoflurane 0.6–2.3 age-adjusted minimal alveolar concentration (MAC) to a target BIS of 45–55. We standardized the perioperative pain management with low dosage of opioid anesthesia (fentanyl: 6–8 μg/kg), non-steroidal anti-inflammatory drugs (NSAIDs), and highly selective α2 adrenergic receptor agonists (Dexmedetomidine) in the present study.

There are four major factors affecting the splanchnic perfusion: (1) intra-abdominal pressure (IAP), (2) position of the patient, (3) carbon dioxide management, and (4) fluid management. In the present study, the intra-abdominal pressure with continuous CO_2_ insufflation was controlled at 10–12 mmHg, the respiratory rate and tidal volume were adjusted to maintain the partial pressure of end-tidal carbon dioxide (P_ET_CO_2_) at 35–45 mmHg. In addition, anti-Trendelenburg position was used for better surgical condition occasionally, but the allowed level of tilt was controlled at 20 degrees, the effect of this position on the hemodynamic parameters was slight. Therefore, the sufficient fluid resuscitation should not be applied for keeping the stability of preload when using anti-Trendelenburg position. If anti-Trendelenburg position affected the hemodynamics, small dosage of phenylephrine hydrochloride (0.1–0.25 μg/kg·min) was used to keep the fluctuations of blood pressure and heart rate within 10% of base value. Furthermore, if the bad surgical conditions could not be improved by anti-Trendelenburg position or higher IAP level, the surgeons preferred to convert to open surgery and the case is to be excluded in this study.

### Postoperative Management

After extubation, all patients were transferred to post-anesthesia care unit (PACU) for further observation. Patient-controlled intravenous analgesia (PCIA) with fentanyl 10 μg/kg, dexmedetomidine 2.5 μg/kg, and Granisetron 6 mg diluted with 0.9% normal saline to a total volume of 100 ml was administered for postoperative pain control. The background infusion was 2 ml/h and the bolus dose was 0.5 ml.

The standard enteral nutrition was started through the nasojejunal tube on postoperative day 1, with a protein:fat:glucose caloric ratio being approximate 20%:30%:50% of one's daily intake.

### Laboratory Analysis

Furthermore, 3 ml of blood was drawn from the cubital vein 24 h before and after surgery and was centrifuged at 1,200 r/min at 4°C for 15 min and stored at −80°C. The level of serum DAO was detected with an enzymatic spectrophotometric assay and the level of serum D-lactate was detected with an enzyme-linked immunosorbent assay according to the instructions of manufacturer (Jiancheng Bioengineering Institute, Nanjing, China).

### 16s rRNA Sequencing of Feces

The postoperative fecal samples were collected in a test tube and immediately refrigerated at −80°C. The 16s rRNA sequencing of feces was entrusted to Suzhou Geneworks Technology Co., Ltd. (Suzhou, China). DNA was extracted using the Magen Hipure Soil DNA Kit (Magen, China) according to the protocol of manufacturer. DNA samples were quantified using a Qubit 3.0 Fluorometer (Invitrogen, Carlsbad, CA, USA). Bacterial 16s rRNA genes of the V3–V4 region were amplified from extracted DNA using the barcoded primers: (5′-CCTACGGRRBGCASCAGKVRVGAAT-3′) and (5′-GGACTACNVGGGTWTCTAATCC-3′). The PCR products were checked for size and specificity by agarose gel electrophoresis and then purified. Finally, high-throughput sequencing was performed using the Illumina MiSeq platform (San Diego, CA, USA). The raw reads were filtered and clustered into operational taxonomic units (OTUs) at the level of 97% similarity using QIIME (Version 1.9.1) and the GreenGene database (Release 13_8_99).

### Analysis for Diversity

Richness estimates and diversity indices (e.g., Chao1, Shannon index, and Simpson index) were calculated using QIIME (Version 1.9.1). A principal component analysis (PCoA) based on the weighted UniFrac distances was conducted to compare all samples by using the R language software (Version 2.15.3, vegan package).

### Study Outcomes

The primary outcomes were the intestinal function recovery and intestinal mucosal barrier: (1) time to flatus and defection; (2) the levels of serum DAO and D-lactate; and (3) the analysis of gut microbiota. The secondary outcomes were: (1) duration of CO_2_ insufflation and surgery; (2) surgical condition scores (five-point scale: 5 points, optimal; 4 points, good; 3 points, acceptable; 2 points, poor; 1 point, extremely poor); (3) time to discharge from the PACU; (4) postoperative pain at 12, 24, and 48 h after surgery using the visual analog pain scores (VAS) (0: no pain; 10: worst pain); (5) postoperative nausea and vomiting; and (6) length of postoperative hospital stay.

### Sample Size Calculation

Sample size was estimated based on the levels of serum DAO and D-lactate. In the preliminary experiment, deep NMBs are more common and better accepted by the surgeons. The design therefore accords with the interest of patients from an ethical point of view. To this end, 1.5:1 randomization between deep and moderate NMB groups was used in this study. Type I error (α) was set at 0.05, and power was set at 80%. Considering a drop-out rate of 10%, a total sample size of 160 patients (96 patients in deep NMB group, 64 patients in moderate NMB group) was required.

### Statistical Analysis

The analyses were conducted using IBM SPSS 20.0 (IBM Corp., Armonk, NY, USA). Normally distributed continuous variables are reported as mean ± SD and were analyzed with two-sided Student's *t*-test. Skewed continuous variables are reported as median (range) and were analyzed with Mann–Whitney *U*-test. Categorical variables are reported as number (*n*) and percentage or rate (%), and χ^2^ test, corrected χ^2^ test, or Fisher's exact test was performed for the analysis. The value of *p* < 0.05 was considered statistically significant for all comparisons.

## Results

**Figure 1:**
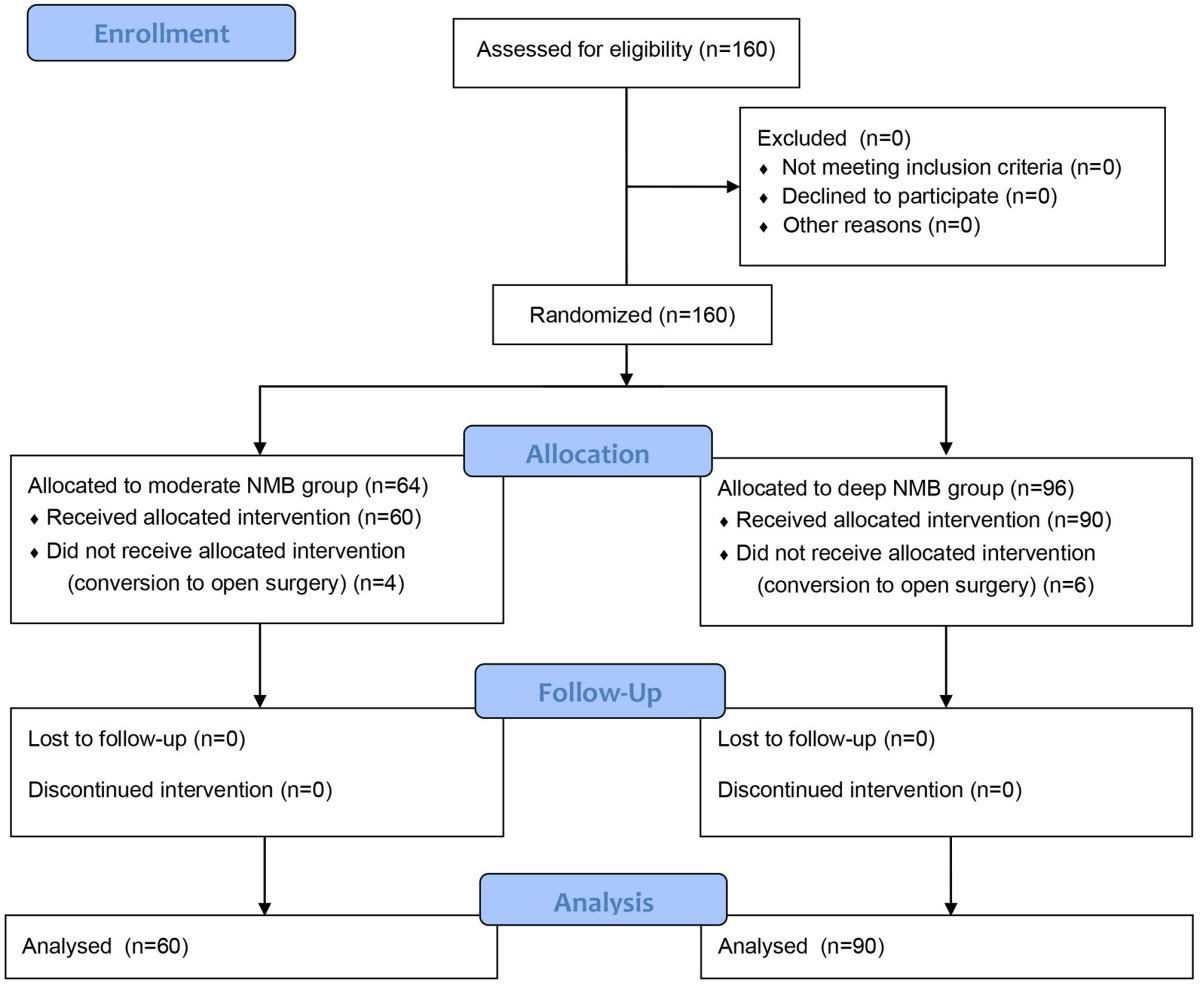
Flowchart of the study.

A total of 160 patients were enrolled in this study. Six patients in the deep NMB group and 4 patients in the moderate NMB group were excluded due to conversion to open surgery (Figure 1). The remaining 150 patients completed the study (90 patients in the deep NMB group and 60 patients in the moderate NMB group). The baseline characteristics were comparable between the two groups (Table 1).

**Table 1:** Baseline characteristics and intraoperative parameters.

	**mNMB (*n* = 60)**	**dNMB (*n* = 90)**
Age (yr)	40–77	41–80
**Sex**, ***n*** **(%)**		
Male	41 (68.3)	59 (65.6)
Female	19 (31.7)	31 (34.4)
BMI kg · m^−2^, mean (SD)	23.7 (2.7)	23.8 (2.7)
**ASA classification**		
I	2 (3.3)	5 (5.6)
II	45 (75.0)	68 (75.6)
III	13 (21.7)	17 (18.8)
**Type of surgery**, ***n*** **(%)**		
Distal gastrectomy	37 (61.7)	46 (51.1)
Proximal gastrectomy	2 (3.3)	2 (2.2)
Total gastrectomy	21 (35.0)	42 (46.7)

*ASA, American Society of Anesthesiologists; BMI, body mass index; dNMB, deep neuromuscular blockade group; mNMB, moderate neuromuscular blockade group; SD, standard deviation*.

As is shown in Table 2, ninety samples in the deep NMB group and sixty samples in the moderate NMB group were analyzed. The average time acquired to flatus after surgery in the deep NMB group was 74 ± 32 h while it took 93 ± 52 h the in moderate NMB group (*p* = 0.006). The serum concentration of D-lactate was statistically decreased compared with that of moderate NMB group (1,164 ± 185 vs. 1,209 ± 224 ng/ml, *p* < 0.001), suggesting that the damage of intestinal mucosa was more severe in the moderate NMB group compared with the deep NMB group. However, there was no significant difference in the level of serum DAO between the two groups (18 ± 4 vs. 23 ± 7 U/L, *p* = 0.220).

**Table 2:** Time to flatus and defecation, and the levels of serum diamine oxidase and D-Lactate.

	**mNMB (*n* = 60)**	**dNMB (*n* = 90)**	***P*-value**
Time to flatus (h), mean (SD)	93 (52)	74 (32)	0.006
Time to defecation (h), mean (SD)	141 (59)	146 (44)	0.702
**Diamine oxidase (U/L), mean (SD)**			
Before surgery	16 (5)	16 (4)	0.931
24 h after surgery	23 (7)	18 (4)	0.220
**D-Lactate (ng/ml), mean (SD)**			
Before surgery	1,037 (186)	1,040 (192)	0.601
24 h after surgery	1,209 (224)	1,164 (185)	<0.001

*dNMB, deep neuromuscular blockade group; mNMB, moderate neuromuscular blockade group; SD, standard deviation*.

**Figure 2:**
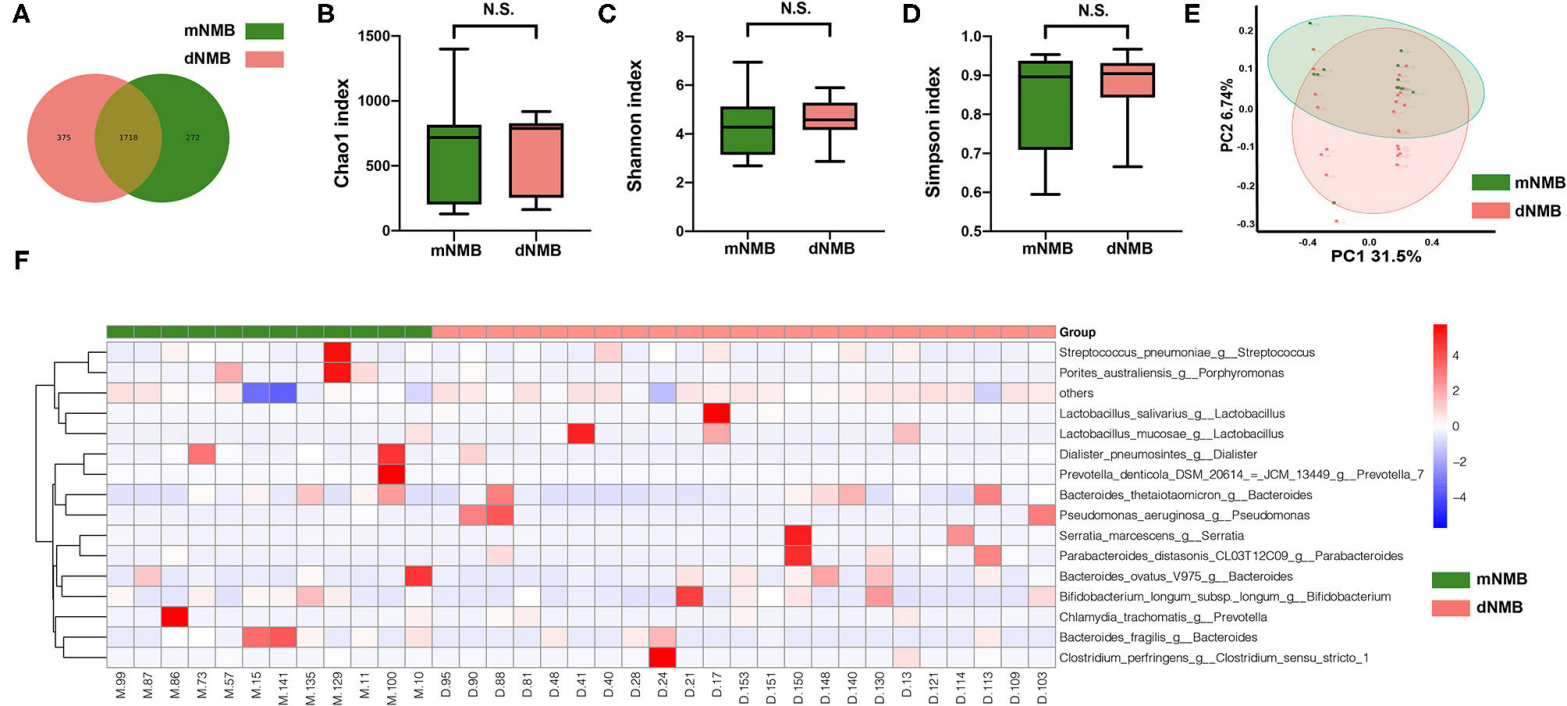
Alpha diversity, Beta diversity, and heatmap of gut microbiota between the deep NMB and moderate NMB group. **(A)** Venn plot; **(B)** Chao 1 index; **(C)** Shannon index; **(D)** Simpson index; **(E)** PCoA of gut microbiota; **(F)** the heatmap at the genus level. dNMB, deep neuromuscular blockade; mNMB, moderate neuromuscular blockade; N.S., not significant; PCoA, principal coordinate analysis.

A total of 35 fecal samples, such as 12 samples in the moderate NMB group and 23 samples in the deep NMB group, were analyzed by 16s rRNA sequencing. The diversity of gut microbiota was more plentiful in the deep NMB group compared with the moderate NMB group (Figure 2A). The Alpha diversity, such as Chao 1, Shannon, and Simpson index, which refers to the diversity of microbiota within a habitat, was similar between the groups (Figures 2B–D). Beta diversity, such as PCoA analysis, indicates the variations of species between habitats. The PCoA analysis plots showed that the dots of deep NMB group were not close to the dots of moderate NMB group (Figure 2E). The heatmap at the genus level was displayed in Figure 2F. In detail, the beneficial bacteria (e.g., *Lactobacillus* and *Bifidobacterium*) were more abundant in the deep NMB group, while the potentially harmful bacteria (e.g., *Dialister*) was more abundant in the moderate NMB group.

**Figure 3:**
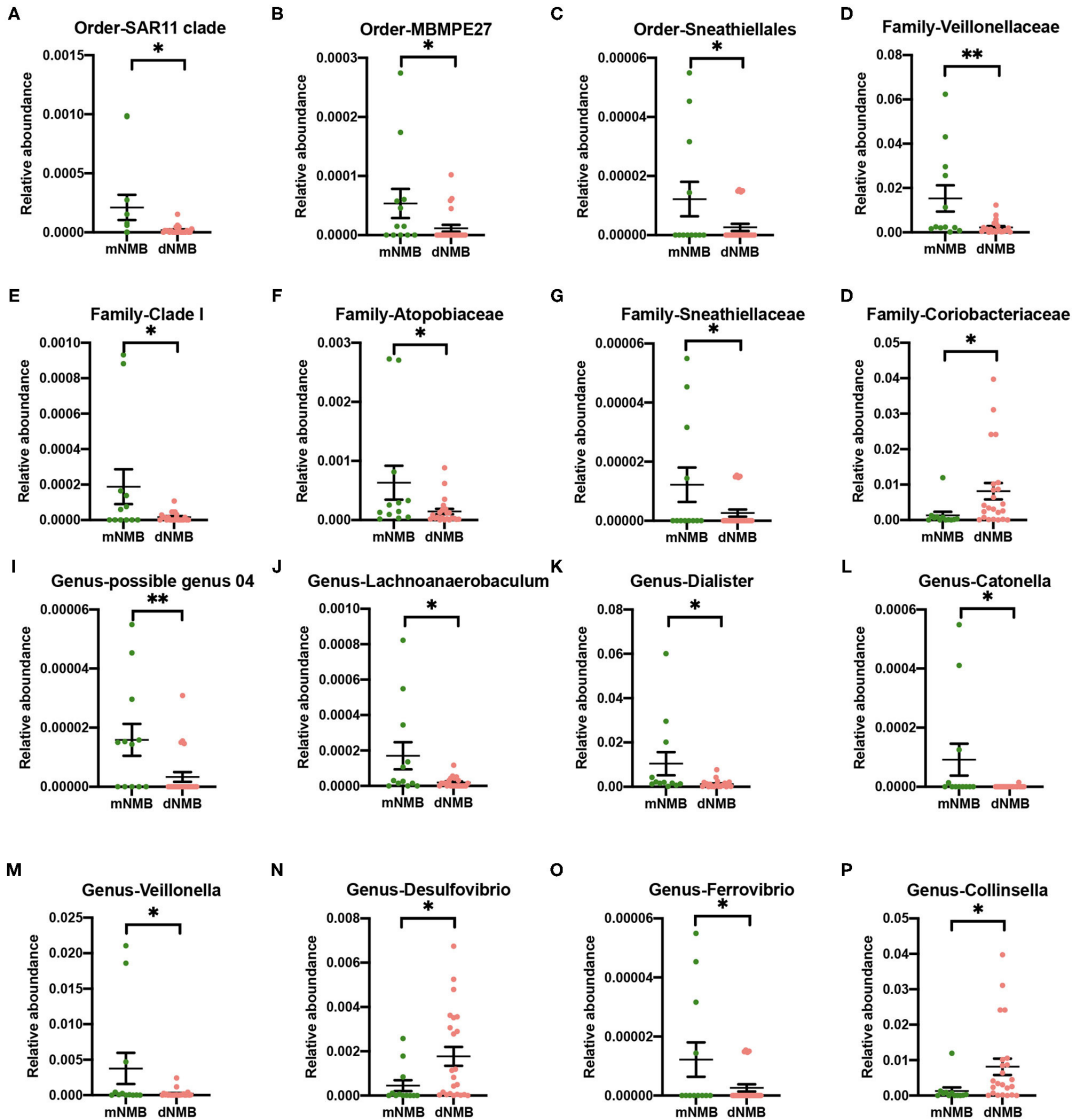
Alterations in the levels of gut microbiota between the deep NMB and moderate NMB group. **(A)** Order *SAR11 clade*; **(B)** Order *MBMPE27*; **(C)** Order *Sneathiellales*; **(D)** Family *Veillonellaceae*; **(E)** Family *Clade I*; **(F)** Family *Atopobiaceae*; **(G)** Family *Sneathiellaceae*; **(H)** Family *Coriobacteriaceae*; **(I)** Genus *possible genus 04*; **(J)** Genus *Lachnoanaerobaculum*; **(K)** Genus *Dialister*; **(L)** Genus *Catonella*; **(M)** Genus *Veillonella*; **(N)** Genus *Desulfovibrio*; **(O)** Genus *Ferrovibrio*; **(P)** Genus *Collinsella*. *p < 0.05, **p < 0.01. dNMB, deep neuromuscular blockade; mNMB, moderate neuromuscular blockade.

The variations of gut microbiota between the groups were functionally relevant. The relative abundance of Family *Coriobacteriaceae*, Genus *Desulfovibrio*, and Genus *Collinsella* was significantly higher in the fecal samples of the deep NMB group (Figures 3H,N,P). In addition, the relative abundance of Order *SAR11 clade, MBMPE27*, and *Sneathiellales*; Family *Veillonellaceae, Clade I, Atopobiaceae*, and *Sneathiellaceae*; Genus *possible genus 04, Lachnoanaerobaculum, Dialister, Catonella, Veillonella*, and *Ferrovibrio* was significantly lower in the deep NMB group (Figures 3A–G,I–M,O).

The surgical condition scores were 3.4 ± 1.1 points in the moderate NMB group, and the scores were 4.8 ± 0.5 points in the deep NMB group (*p* < 0.001). The duration of the surgery in the deep NMB group (171.0 ± 30.8 min) was significantly reduced compared with the moderate NMB group (184.0 ± 33.7 min, *p* = 0.017). There were no statistical differences in the length of PACU stay and the postoperative hospital stay between the two groups. Meanwhile, postoperative VAS pain scores at 12, 24, and 48 h after surgery were similar in the two groups. Postoperative death was not observed in both groups before the patient was discharged from the hospital (Table 3).

**Table 3:** Intra- and postoperative parameters.

	**mNMB (*n* = 60)**	**dNMB *(n* = 90)**	***P*-value**
**Intraoperative**			
Surgical condition scores, mean (SD)	3.4 (1.1)	4.8 (0.5)	<0.001
5, *n* (%)	10 (16.7)	77 (85.6)	
4, *n* (%)	20 (33.3)	9 (10.0)	
3, *n* (%)	23 (38.3)	4 (4.4)	
2, *n* (%)	6 (10.0)	0 (0.0)	
1, *n* (%)	1 (1.7)	0 (0.0)	
Total time of surgery (min), mean (SD)	184 (33.7)	171 (30.8)	0.017
Total time of CO_2_ insufflation (min), mean (SD)	155 (31.7)	141 (34.4)	0.010
**Postoperative**			
Duration of PACU stay (min), mean (SD)	10.06 (7.8)	8.9 (5.5)	0.349
**VAS scores, mean (SD)**			
12 h after surgery	4.0 (1.4)	3.8 (1.5)	0.551
24 h after surgery	3.2 (1.3)	2.9 (1.2)	0.225
48 h after surgery	1.7 (1.0)	1.6 (1.1)	0.748
Hospital stays after surgery (day), mean (SD)	10.1 (7.8)	8.9 (5.4)	0.349
PONV, *n* (%) ONV	7 (11.7)	8 (8.9)	0.30
Death, *n* (%)	0 (0.0)	0 (0.0)	0.99

*CO_2_, carbon dioxide; dNMB, deep neuromuscular blockade group; mNMB, moderate neuromuscular blockade group; PACU, post-anesthesia care unit; PONV, postoperative nausea and vomitting; VAS, visual analog scale; SD, standard deviation*.

## Discussion

This study demonstrated that deep NMB in laparoscopic gastrectomy could help to cut down the time to postoperative flatus and reduce the damage to intestinal mucosal barrier and gut microbiota. The beneficial bacteria were better preserved in the deep NMB group. In addition, deep NMB created better surgical conditions and shortened the average duration of the surgery.

Laparoscopic surgeries have been the main treatment option for abdominal surgeries and have been widely accepted with minimal incision compared with traditional open surgeries. But the indispensable and continuous insufflation of CO_2_ during laparoscopic surgeries results in the artificial intra-abdominal hypertension, which would aggravate the perfusion of intra-abdominal organs (14). Studies reported a decrease of blood flow in both the superior mesenteric artery and hepatic portal vein under the CO_2_ pneumoperitoneum of 14 mmHg (14, 15). The ischemic injury may cause deleterious effects to the intestinal mucosal barrier and postpone the postoperative recovery (16). In addition, studies have shown the benefits of deep NMB in optimizing surgical fields and reducing the morbidity of unexpected muscle retractions in major surgeries. Deep NMB is beneficial for the recovery of intestinal recovery and postoperative rehabilitations (10). But to date, the mechanisms of deep NMB on enhancing the recovery of postoperative intestinal function after laparoscopic gastrectomy are not clear.

In this study, time to flatus after surgery was significantly reduced in the deep NMB group (Table 2). However, mainly owning to the insufficient food intake, time to defection was similar between the groups. To evaluate the influence of different depth of NMB on the integrity of intestinal mucosal barrier, we tested the serum levels of DAO and D-lactate. A previous study showed that a receiver operating characteristic (ROC) analysis revealed that the sensitivity of D-lactate was 0.91, but the specificity was 0.70. The accuracy of D-lactate was very high, and that the areas under the curve (AUC) of the biomarker was 0.84. The sensitivity of DAO was 0.25, and its specificity was 0.92 (2). The present study showed that the increase of serum D-lactate was significantly lower in deep NMB group compared with in moderate NMB group, though the two biomarkers were increased after surgery among all the patients, suggesting that deep NMB was beneficial to protect the integrity of intestinal mucosal barrier. The relatively high levels of serum DAO and D-lactate observed in the moderate NMB group were consistent with the prolonged time to flatus after surgery. Therefore, we consider that deep NMB helps to enhance the postoperative the recovery of bowel movement by causing less damage to the intestinal mucosa in laparoscopic gastrectomy.

The important role of gut microbiota in human health and diseases, such as inflammatory or immune disorders, has been gradually recognized (17). Given the fact that alterations of the peritoneal fluid and the peritoneal microcirculation caused by the insufflation of CO_2_ during the surgery, gut microbiota would transform to a more virulent phenotype (18–20). The alterations of normal gut microbiota could aggravate intestinal inflammation and prolong postoperative recovery (21, 22). Alpha and Beta diversity are effective indicators to reflect the within-habitat and between-habitat diversity of gut microbiota. This study showed that the Alpha diversity exhibited few differences (Figure 2). Interestingly, the Beta diversity demonstrated that the dots of deep NMB group were separated from those of moderate NMB group (Figure 2). The heatmap indicated that the relative abundance of several bacteria at the genus level was significantly different between the groups. The *Lactobacillus* and *Bifidobacterium* were more abundant in the deep NMB group, whereas the relative abundance of genera *Dialister* were much higher in the moderate NMB group. The *Lactobacillus* and *Bifidobacterium* are generally beneficial bacteria and are essential to enhance the intestinal microecology (23). However, the increased relative abundance of genera *Dialister* was observed among patients suffering from constipation (24, 25). The 16s rRNA sequencing demonstrated that the levels of Genus *Desulfovibrio* were significantly higher in the deep NMB group than those in the moderate NMB group. It has been reported that the levels of Genus *Desulfovibrio* were negatively correlated with intestinal inflammation (26). This may be a key bacterium to relieve the intestinal inflammation in moderate NMB group.

It has been proved that deep NMB could provide better surgical conditions with the same intra-abdominal pressure and reduce the incidence of muscular contractions (27). It is essential to be fully paralyzed, especially in laparoscopy, as the sudden body movement would result in hemorrhage or severe organ damages if it was in critical steps (28, 29). In this study, the incidence of muscle contractions was less in the deep NMB group than in the moderate NMB group. The surgeons were much more satisfied with the surgical conditions with deep NMB, which was consistent with previous studies (30). The relatively roomy surgical field could provide a better pre-judgment to probable situations and would improve the surgical process (31).

Postoperative pain and the opioid treatment are controversial factors prolonging the recovery of intestinal function. The side effect of opioid is constipation. So, we standardized the perioperative pain management with low dosage of opioid. A previous study found that the patients in the deep NMB group suffered from a lower intensity of postoperative abdominal pain after laparoscopic colorectal resection within the following 48 h after the surgery (10). However, low insufflation pressure with deep NMB did not result in reducing the pain in laparoscopic cholecystectomy (32). In this study, there was no significant difference in postoperative pain between the groups owing mainly to the relatively minimally invasive surgical incision and complete postoperative analgesia in this study.

There are some improvements that could be applied in the following investigations. To detect a convincing correlation between intra-abdominal pressure and histologic lesions, a biopsy of intestinal tissue was more persuasive. But it was unethical in humans to get extra intestinal tissue. In addition, splanchnic perfusion is very important for laparoscopic surgery, the present study ensured the splanchnic perfusion by controlling the IAP at 10–12 mmHg and PetCO_2_ at 35–45 mmHg, but we did not collect the data on hemodynamics and monitor the splanchnic perfusion. More studies should be carried out and better methods should be applied to monitor the parameters, such as intraoperative ultrasound in further studies. Moreover, the gut varies even in the same surgical procedure, and this could affect the findings. In the study, methods to evaluate the surgical difficulties were insufficient. Meanwhile, the mechanisms of the gut microbiota on health and diseases remain to be determined and the effect of perioperative probiotics remains to be identified in the future.

In summary, deep NMB, compared with moderate NMB, helps to preserve the intestinal function after laparoscopic gastrectomy with less damage to intestinal mucosa and gut microbiota. Therefore, deep NMB is worth taking into consideration for patients undergoing laparoscopic gastrectomy in terms of the effect of intestinal protection.

## Data Availability Statement

The original contributions presented in the study are publicly available. This data can be found here: https://www.ncbi.nlm.nih.gov/bioproject/PRJNA788087.

## Ethics Statement

The studies involving human participants were reviewed and approved by First Affiliated Hospital of Nanjing Medical University, Nanjing, China (No: 2018-SR-336). The patients/participants provided their written informed consent to participate in this study.

## Author Contributions

HH, CL, and CY: conceived and designed the experiments. HH, LZ, YY, and HX: performed the experiments. SL and ZX: data analysis and interpretation. HH, LZ, CL, and CY: manuscript preparation. All authors contributed to the article and approved the submitted version.

## Funding

This work was supported by the Wu Jieping Medical Foundation, China (No. 320.6750.18180), the Jiangsu Province Special Program for Young Medical Talent (No. QNRC2016587), the National Natural Science Foundation of China (Nos. 81703482, 81901100, and 81974171), the Natural Science Foundation of Jiangsu Province, the Innovative and Entrepreneurial Team of Jiangsu Province (No. JSSCTD202144), and Natural Science Foundation of Jiangsu Province (No. BK20211382).

## Conflict of Interest

The authors declare that the research was conducted in the absence of any commercial or financial relationships that could be construed as a potential conflict of interest.

## Publisher's Note

All claims expressed in this article are solely those of the authors and do not necessarily represent those of their affiliated organizations, or those of the publisher, the editors and the reviewers. Any product that may be evaluated in this article, or claim that may be made by its manufacturer, is not guaranteed or endorsed by the publisher.
